# me98 is a new allele of rad-54

**DOI:** 10.17912/micropub.biology.000108

**Published:** 2019-04-26

**Authors:** Baptiste Roelens, Karl A Zawadzki, Anne M Villeneuve

**Affiliations:** 1 Departments of Developmental Biology and Genetics, Stanford University School of Medicine

**Figure 1.**
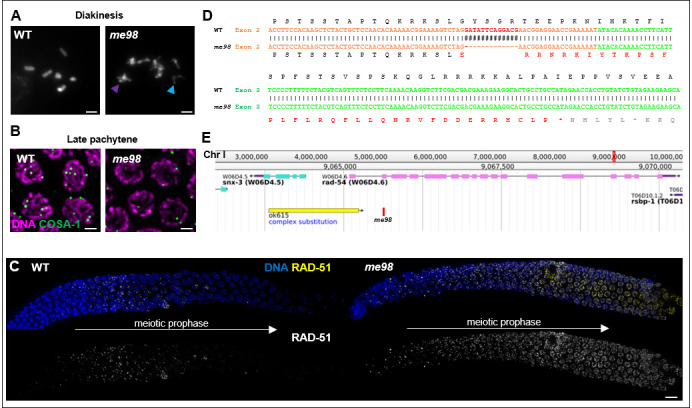
DAPI stained chromosomes in oocytes of the indicated genotype at diakinesis, the last stage of meiotic prophase; while six pairs of attached homologs are detected in wild-type animals, chromosome structural defects such as fragments (blue arrowhead) or partially decondensed chromosomes (purple arrowhead) are observed in the *me98*mutant. **B.** Detection of the crossover site marker GFP::COSA-1 in WT (left) and *me98* mutant germ-cells (right). Scale bars on panels A and B represent 2µm. **C.** Detection of the recombinase RAD-51 in the region of the gonad corresponding to early to middle stages of meiotic prophase in WT (left) and *me98* mutant (right) worms. RAD-51 foci are transiently detected in wild-type animals in early prophase as DNA breaks are formed and repair progresses. In contrast, in *me98* mutants, RAD-51 foci accumulate and remain at high levels. Scale bar represents 10µm. **D.** Position and nature of the *me98* deletion, located in the second exon of the *rad-54* coding sequence, and its consequences on the encoded protein product. **E.** Screenshot of genome browser centered on the *rad-54* locus. Positions of the previously described *rad-54* allele, *ok615*, and the newly identified me98 mutation are represented respectively as a yellow and a red bar.

## Description

We isolated the *me98* mutant in a genetic screen for *C. elegans* mutants with an altered number of GFP::COSA-1 foci, which mark the sites of crossovers in wild-type *C. elegans* germ cells (Rosu *et al.* 2013). After multiple rounds of outcrossing, we confirmed that the *me98* mutant is defective in meiotic prophase as *i*) chromosomes in diakinesis oocytes appear partially decondensed and structurally compromised (Fig. 1A), *ii*) *me98* fails to form the six GFP::COSA-1 foci observed in wild-type late pachytene meiocytes (Fig. 1B) and *iii*) *me98* accumulates RAD-51 foci during the course of meiotic prophase (Fig. 1C). Further, 100% of eggs laid by *me98* mutant hermaphrodites are inviable. These defects are reminiscent of those caused by the previously-described *rad-54(ok615)* mutation (Mets and Meyer 2009), and sequencing of the *rad-54* locus in *me98* mutants revealed the presence of a 13bp deletion in the second exon of the annotated transcript (I:9065652 to I:9065664 of WS269). This lesion creates a frameshift that would result in premature termination of translation in the third exon (of seventeen) of the predicted transcript (Fig. 1D), suggesting that it is likely a null allele. Of note, the previously described *rad-54* loss-of-function allele, *ok615*, is an insertion/deletion that also affects the neighboring gene *snx-3* (Fig 1E). As gonads of *rad-54(me98)* mutants appear overall healthier than those in the *ok615* mutant, *me98* could be a valuable tool to analyze the specific function of *rad-54*.

## Methods

Cytology: Immunofluorescent detection of GFP::COSA-1 and RAD-51 was performed as described in (Martinez-Perez and Villeneuve 2005) using a mouse anti-GFP antibody (Sigma-Aldrich #11814460001) and a rabbit anti-RAD-51 antibody (Colaiacovo *et al.* 2003).

## Reagents

Strains:

AV727: *meIs8[pie-1p::gfp::cosa-1 + unc-119(+)] II;ltIs37[pie-1p::mCherry::his-58 + unc-119(+)] IV;ltIs38[pie-1p::gfp::ph(PLC1delta1) + unc-119(+)]*

AV762: *rad-54(me98)/hT2[qIs48] (I;III);meIs8[pie-1p::gfp::cosa-1 + unc-119(+)] II;ltIs37[pie-1p::mCherry::his-58 + unc-119(+)] IV;ltIs38[pie-1p::gfp::ph(PLC1delta1) + unc-119(+)]*
